# Clinical Variables Associated with Physician-Driven Inclusion in a Special Management Program for Complex Patients †

**DOI:** 10.3390/jcm15010202

**Published:** 2025-12-26

**Authors:** Vered Mintzer, Eugene Merzon, Ariel Israel, Shai Ashkenazi, Ayala Blau, Eli Magen, Shlomo Vinker, Ilan Green, Avivit Golan-Cohen

**Affiliations:** 1 Adelson School of Medicine, Ariel University, Ariel 40700, Israel; 2 Leumit Health Services, Tel Aviv-Yafo 64738, Israelsvinker@leumit.co.il (S.V.);; 3 Department of Nursing, Faculty of Health Science, Ariel University, Ariel 40700, Israel; ayalabl@ariel.ac.il; 4 Department of Epidemiology and Preventive Medicine, School of Public Health, Gray Faculty of Medical & Health Sciences, Tel Aviv University, Ramat Aviv, Tel Aviv 6997801, Israel; 5 Department of Medicine A, Assuta Ashdod University Medical Center, Ben Gurion University of the Negev, Beer Sheva 8410501, Israel; allergologycom@gmail.com; 6 Gray Faculty of Medical & Health Sciences, Tel Aviv University, Ramat Aviv, Tel Aviv 6997801, Israel

**Keywords:** complex patients, multiple chronic conditions, team care management

## Abstract

**Background/Objectives:** The increasing rate of complex patients with multiple chronic somatic and/or mental disorders in modern medicine is challenging, necessitating special management programs. The aim of the present study was to identify clinical variables and the use of health services associated with the primary-physician-driven inclusion of complex patients in the “Team Management for Complex Patients” (TMCP) special program. **Methods:** Using validated electronic medical records of a nationwide health maintenance organization, a case–control study was performed. The study compared parameters before enrollment of complex patients included in the TMCP program with those of complex patients during the same time period who were not included, and were matched using a propensity score for age, sex, socioeconomic status, place of residence, ethnicity, smoking status, physical activity, and the balance before the day of enrollment for the major body measurements and laboratory results. **Results:** The control group was well-balanced, except for the South region and no physical activity. Several respiratory, cardiac, gastrointestinal, neurological, inflammatory and autoimmune diseases were significantly more common among patients included in the TMCP program than among those not included. Complex patients included in the program presented significantly higher previous rates of attending outpatient urgent care centers, visiting hospital emergency departments, hospitalization, and medication use. **Conclusions:** Although limited by subjective inclusion criteria and potential confounding, the present comparative study identified clinical variables associated with the identification of complex patients for enrollment into a special managed program. These associations may inform future work to develop and validate criteria to support physician decision-making in selecting complex patients for managed programs and designing healthcare resources for patients who need them most. We currently meticulously follow the outcomes of the patients included in the special managed program.

## 1. Introduction

### 1.1. Complex Patients

The term complex patients usually refers to individuals who have multiple significant chronic disorders, such as diabetes mellitus, hypertension, chronic respiratory disease, ischemic heart disease, and chronic renal diseases [1,2,3]. Therefore, their challenging healthcare needs extend beyond the standard medical care [2,4,5]. Complexity in these patients spreads beyond the number of chronic diseases and is shaped by dynamic interactions among health problems, treatments, patient behaviors, and social circumstances—factors that complicate planning, coordination, and outcomes [3,6,7,8]. Accurately identifying complex patients in primary care is crucial for optimizing outcomes and resource allocation, as these individuals often present with multifaceted medical, psychological, and social needs that exceed the scope of standard care models. While complexity is typically defined by clinical criteria, such as multimorbidity and polypharmacy, physicians frequently rely on their experience, labeling patients as ‘difficult’ when faced with diseases, behaviors or circumstances that complicate care delivery [9]. This overlap between perceived difficulty and medical complexity underscores the need for structured approaches to patient selection for specialized management programs [10]. Studies have identified ~26% of primary care patients as complex, with mental health, substance use, and care coordination being major contributors [11]. These patients often present polypharmacy, functional disability, psychiatric disorders, cognitive impairment, low health literacy, fragmented social support and limited access to resources, which result in poor medical outcomes, like frequent and multiple hospitalizations [6,8,12]. With the global aging population and the increased survival of patients with chronic diseases in the advanced modern medicine, the rates of complex patients are increasing [13]. Understanding this multidimensional complexity is essential for patients’ identification and designing effective care models.

### 1.2. Management of Complex Patients

Managing complex patients is costly and poses significant challenges for public healthcare systems [2,3,11,14]. While primary care physicians often serve as case managers [3,5,15,16], these patients require a multidisciplinary approach involving coordinated input from various professionals [8,12,17,18]. Recent evidence emphasizes that care should be patient-centered, individualized, and holistic, addressing not only medical needs but also patient preferences, mental health, and social determinants [6,19,20]. Rigid application of disease-specific guidelines often leads to fragmented care and may even harm this vulnerable population [21,22].

### 1.3. Programs for the Management of Complex Patients

Several programs have been developed to improve outcomes for complex patients, focusing on multidisciplinary collaboration [18,23], communication [24], defragmentation [21], and informal care [17], while supporting primary care physicians [16,25]. Community-based interventions that integrate holistic planning and team-based care have demonstrated benefits such as reduced mortality, improved cognitive function, and enhanced quality of life [26,27,28,29].

The Team Management for Complex Patients (TMCP) program is a proactive, multidisciplinary intervention supported by the Israeli Ministry of Health (MoH). The definition of complex patients for the TMCP program was individuals with two or more chronic medical conditions (such as diabetes mellitus, ischemic heart diseases, chronic obstructive pulmonary disease, chronic renal failure), who were identified by their primary care physicians as presenting significant challenges for appropriate healthcare delivery. The program’s goals are to identify complex patients with multiple chronic conditions in ambulatory care and to provide personalized, coordinated management through a multidisciplinary team. This team includes primary care physicians, nurses, social workers, administrative coordinators, and other healthcare professionals. The program is structured and benchmarked according to strict MoH guidelines.

Leumit Health Services (LHS) is a large, nationwide Health Maintenance Organization (HMO) in Israel that provides healthcare services to over 725,000 members of diverse geographic, socioeconomic, and ethnic backgrounds. Since 2020, LHS has implemented the special TMCP interventional program, with eligible individuals defined as patients with at least two chronic diseases. Primary care physicians were instructed on the definition and characteristics of complex patients and the TMCP by a specific learning model. The inclusion of eligible patients in the program was according to the decision of their primary care physicians, who have followed the patients and have a comprehensive view of the patients’ medical complexity and special needs for healthcare services. Not all patients with multiple chronic conditions have been included in this program.

### 1.4. Aims of the Study

The aim of this study was to describe clinical characteristics and prior healthcare utilization associated with physician-driven enrollment of patients with ≥2 chronic conditions into the TMCP program. We compared pre-enrollment characteristics of enrolled patients to propensity score-matched controls who met the chronic-disease criterion but were not enrolled during the same period. The primary care physician underwent a learning session and then enrolled their patients into the TMCP according to their complexity and the need for special healthcare services. The importance of careful population selection for complex care interventions was previously highlighted by the widely varied patient cohorts included in various definitions of complex patient populations [7].

## 2. Methods

### 2.1. Study Design

A real-world, population-based, case–control study was conducted. The study examined the clinical characteristics and emergency healthcare use of complex patients who have been included in the TMCP program compared with patients with multiple chronic disorders who were not enrolled in the program during the same time period as the cases. The controls were matched to the enrolled patients (“cases”) using a propensity score matching method, which considered multiple variables detailed below. The study protocol was approved by the Ethic and Research Committee of LHS (approval number: LEU-0013-24). The inclusion of patients in TMCP program is based on their agreement.

### 2.2. Data Source and Study Population

Demographic data, clinical information, and laboratory results were extracted from the electronic medical records (EMRs) of LHS. Comprehensive LHS EMRs integrate information on patient socio-demographics, clinical encounters, laboratory results, visits to ambulatory urgent care centers of LHS and hospital emergency departments, hospitalizations, prescription records, and medications purchased. The validity of the database in this registry has been previously established [30].

The cases were all individuals with two or more chronic medical conditions (such as diabetes mellitus, ischemic heart diseases, chronic obstructive pulmonary disease, and chronic renal failure), who were identified by their primary care physicians as presenting significant challenges for appropriate healthcare, and were included in the TMCP program by their primary care physicians from 1 February 2020 until 15 October 2024. The control group was patients with multiple chronic diseases who were not included in the program and individually matched for sociodemographic variables that are known to affect patients’ complexity, namely age, sex, socioeconomic status (SES), geographic region, smoking status, engagement in physical activity, and ethnic sector. Each control was assigned the same index date as the enrollment date of its matched case to ensure temporal alignment. In order to concentrate on clinical variables associated with inclusion in the program, we also attempted to match for compliance: controls that were similarly balanced before enrollment were selected by matching also for the last measurement before the date of enrollment regarding body mass index (BMI), underweight (BMI < 18.5 kg/m^2^), overweight (BMI ≥ 25 kg/m^2^), obesity (BMI ≥ 30 kg/m^2^), systolic and diastolic blood pressure; and the results of hemoglobin A1C, serum glucose, serum lipids and renal function tests. Hemoglobin A1C level was used as a proxy for compliance, which is a critical factor in complexity management. Polypharmacy was defined as the purchase of five or more distinct chronic medication classes during the 10-year period prior to the index date. The case and control groups were then compared regarding underlying chronic diseases, medication consumption, hospitalizations, emergency department visits, and urgent care centers attendance during the 10 years prior to enrollment.

### 2.3. Statistical Analysis

Statistical analyses were performed using R software version 4.0.2 (R Foundation for Statistical Computing, Vienna, Austria). All tests were two-sided, with a significance threshold set at 0.05. Mean ± standard deviation (SD) and median and interquartile range (IQR) were calculated, as appropriate. Comparisons of sociodemographic and clinical characteristics between the patients included in the TMCP (cases) and those that were not included (controls) were conducted using the *t*-test for continuous variables and Fisher’s exact χ^2^ test for categorical variables, based on data distribution and variable type. Logistic regression analysis was employed to assess the independent likelihood of being included in the interventional management program compared to the controls. Effect sizes were expressed as odds ratios (ORs) with 95% confidence intervals (CIs). To adjust for multiple comparisons, the Benjamini–Hochberg procedure was applied to control the false discovery rate (FDR).

Propensity score specification and matching

We estimated a propensity score (PS) via logistic regression including age, sex, socioeconomic status (continuous and categorical), geographic region (Center, North, South, Jerusalem), ethnic sector, smoking status, physical activity (4-level), BMI, systolic/diastolic blood pressure, HbA1c, fasting glucose, LDL, HDL, triglycerides, creatinine/eGFR, and urine albumin-to-creatinine ratio. One-to-one nearest-neighbor matching without replacement was performed with a 0.2 SD caliper on the PS logit. Covariate balance was evaluated using SMDs with a target <0.10.

Sensitivity analyses

To assess robustness, we performed sensitivity analyses using a 5-year lookback period for utilization and medication history, excluding the top 2% of patients with extreme emergency department visits or hospitalizations, and stratified results by age group (≥60 years vs. <60 years). These analyses were conducted using the same statistical approach as the primary analysis.

Propensity score matching and covariate balance assessment were performed (Supplementary Table S1). Variables retained in the multivariable model and sensitivity analyses are detailed in Supplementary Tables S2 and S3.

## 3. Results

### 3.1. Matched Sociodemographic, Physical Measurements, and Laboratory Results

Table 1 presents the matched demographics, physical measurements, and laboratory indicators of the case and control groups, demonstrating a very good matching, except for the South region and no physical activity. Demographics indicated that males predominated in both groups (55.8%), with a similar mean age of ~65 years, 60% from the general sector, 74% non-smokers and a mean SES score of 8. There were mild differences between the cases and the controls in the rates of living in the south (21.7% vs. 24.7%) and with no engagement in physical activity (52.7% vs. 48.4%). Regarding physical measurements, in both groups the mean BMI was 29.4 kg/m^2^, with similar rates of underweight, overweight, and obesity (~1.6%, 34% and 41%, respectively), and no significant differences in the means of systolic or diastolic blood pressures. Regarding the laboratory results before the date of enrollment, hemoglobin level was mildly lower in the case than in the control group (13.3 vs. 13.6 g/dL, both within normal range), with no significant differences in hemoglobin A1C, serum glucose, renal function tests, and lipid levels. Details are presented in Table 1.

### 3.2. Underlying Chronic Diseases

Table 2 presents the chronic diseases encountered in the cases and control groups. As is evidenced, multiple chronic conditions—respiratory, cardiac, gastrointestinal, neurologic, inflammatory, and autoimmune diseases—were more common among the patients included in the TMCP program than in those who were not included. Diseases with highest odds rations (numbers in parenthesis) between the groups were liver cirrhosis (2.56), epilepsy (2.11), fibromyalgia (1.91), bronchial asthma (1.84), cardiomyopathy (1.79), connective tissue disease (1.69), cognitive disorders (1.61), previous falls (1.60), osteoporosis (1.59), and atrial fibrillation (1.56). Nearly all differences, excluding cerebrovascular accidents and depression, reached statistical significance. By adjusting the control and case groups for hemoglobin A1C, in order to control for compliance, diabetes was neutralized. Numerical details of the rates of chronic diseases in the two groups are given in Table 2.

### 3.3. Chronic Medication Usage

Table 3 demonstrates the main classes of medications purchased by the case and control groups during the 10-year period before the date of enrollment in the special management program or in the control group. As can be seen, all medications examined were used significantly more commonly by the cases than by the controls, with most odds ratios above 1.5. Cases used significantly more insulin, glucagon-like peptide-1 (GLP-1) receptor analogs, antibiotics, steroids (systemic and local), opioids, non-steroidal anti-inflammatory agents (NSAIDs), and medications for respiratory, cardiac, gastrointestinal, and psychiatric disorders. Precise numerical details are given in Table 3.

### 3.4. Hospitalizations and Attendance of Urgent Care Services

Table 4 presents the hospitalizations and visits to the urgent care service of the case and control groups during the 10-year period prior to the date of enrollment in the special management program and in the control group during the same time period. The urgent care services included outpatient urgent care centers (UCCs) of the HMO and emergency departments at hospitals. As can be seen, visits to the urgent care ambulatory services, emergency departments and hospitalizations were more common among the patients included in the TMCP program than in those who were not included. Variables with highest Odds rations (numbers in parenthesis) between the groups were in visit to an outpatient UCC (2.10), hospitalization for ≥1 day in a geriatric-internal medicine department (2.39), hospitalization in internal medicine department (2.11), hospitalization in internal medicine for ≥4 days (2.00), hospitalization in the geriatric rehabilitation center (1.93), a visit to a hospital emergency department (1.83), and hospitalization in an intensive care unit (1.53). All differences reached statistical significance. Numerical details of the rates in the two groups are given in Table 4.

Covariate balance after propensity score matching met the predefined threshold, with all standardized mean differences below 0.10 (Supplementary Table S1). Independent predictors of inclusion in the TMCP program were identified using a parsimonious multivariable logistic regression model, which highlighted polypharmacy, prior emergency department visits, previous hospitalizations, and selected chronic conditions such as COPD, fibromyalgia, and osteoporosis (Supplementary Table S2). Sensitivity analyses—including alternative handling of missing data (Supplementary Table S3), a 5-year lookback period, exclusion of high-utilization outliers, and age stratification—yielded results consistent with the primary analysis.

## 4. Discussion

### 4.1. New Findings

The present study identified several precise clinical variables and use of health services amongst complex patients that were associated with real-life inclusion in a special managed intervention program, compared to complex patients who were not included in the program and matched for sociodemographic variables and laboratory results before enrollment. This unique methodology controlled sociodemographic variables and compliance, known as related to patients’ complexity. The associated variables that were identified included several underlying chronic medical conditions, multiple medication use, outpatient urgent care centers attendance, emergency department visits, and hospitalizations. These findings documented that certain chronic diseases, such as bronchial asthma, osteoporosis, epilepsy, and fibromyalgia, which are sometimes not perceived as “complex” or “severe”, were significantly more prominent in the patients who had been included in the intervention program. The allocation of patients with these diagnoses to the special managed intervention program probably reflects the identification of patients who were suitable for the TMCP by the primary care physician, who witnessed that these diseases require frequent needs for medical care, as known parameters for complex patients [3,5,12,31,32]. On the other hand, oncological diagnoses were not identified as a parameter for inclusion in the special program, likely because the framework and support provided for managing these patients in our health system are comprehensive and satisfactory—typically offered in secondary or tertiary medical centers. Uncontrolled diabetes and high HbA1C are well-known markers of complexity. By matching for HbA1C to control for compliance, we likely neutralized diabetes mellitus which is obviously an important complexity-related disease. This was necessary for our study design, which aimed to evaluate other, less obvious factors associated with complex patients.

Indeed, complex patients are those with multiple chronic somatic and/or mental disorders that often present polypharmacy, functional disability, cognitive impairment, and poor medical outcomes, thus increased visits in ambulatory urgent care centers and emergency departments, and hospitalization admissions [16,33,34,35]. This study also documented that polypharmacy was associated with inclusion in the program, as polypharmacy and its consequences that were witnessed by the primary care physician likely resulted in selection for the special program, as it reflected a complexity-related variable. It should be emphasized that the selection of the complex patients for the special TMCP intervention program was performed according to the perception of the primary care physicians of these patients.

### 4.2. Implications of the Study Results

The elucidation by the present comprehensive study of the clinical variables and parameters of healthcare services used, that were significantly associated with inclusion of complex patients in a special program, is of importance for the primary care physician and also for management teams of HMOs and other healthcare systems. The rising rates of complex patients in modern medicine, due to the increased survival of patients with chronic diseases, and the special needs of these patients, present a significant challenge to healthcare systems [2,8,11,13,14]. In most healthcare organizations, the primary care physician is the case manager of the complex patients [3,5,16], but a multidisciplinary team-approached medical care is obviously required [12,17,18]. This led to the establishment of special programs to ensure the administration of optimal healthcare for the complex patients, with a multidisciplinary team approach and holistic patient care [6,18,20,23]. Choosing the most appropriate patients in need of the special programs is crucial, especially as the precise definitions of complex patient populations vary considerably among various patient cohorts [7]. The identified variables can therefore aid primary care physicians in designating complex patients to the special programs. These will also assist the management teams of healthcare organizations in planning the special intervention programs for complex patients for efficient allocation of health resources.

### 4.3. Relation to Previous Studies

To the best of our knowledge, this is the first scientific report that applies propensity score for matching complex patients that had been included by the primary care physician in a special multidisciplinary program, and a control group of complex patients that had not been included, and then comprehensively identified clinical variables that were associated with the inclusion. However, our findings are generally in concert with previous studies that have attempted to develop models to identify complex patients and describe their severity [3,5,35,36].

A cluster analysis of complex chronic patients in Spain was conducted to characterize patients’ sub-groups based on clinical judgment, leading to the identification of a high-risk stratum in terms of admissions and life-threatening diseases [3]. Another study evaluated physician-defined complexity of patients by predicting high health system utilization rates, such as emergency room visits and hospital admissions [35]. The INTERMED Complexity Assessment Grid (adult version) was used to assess the “biopsychosocial complexity” in primary care by interviews that synthesize data based on four health-related domains: biological, psychological, social, and health system, with numerical scoring for each domain [5]. This tool demonstrated good feasibility and validity, although it was cumbersome and challenging to implement in practice [3]. Efforts to develop population segments based on primary health care needs have also been undertaken, incorporating patient complexity and vulnerability to create categories ranging from stable patients to those with multi-morbidity and complex needs [36]. A cross-sectional study identified the following factors as associated with patients’ complexity: low socioeconomic status, morbidity issues, and higher healthcare utilization [8]. Several studies have highlighted the central role of the primary care physician in defining complex patients, although an aid is needed for precise definitions, especially of variables related to patients’ complexity [5,7,16,18,28,37]. Management should be patient-centered, with consideration of their preferences [31,38,39].

### 4.4. Strengths and Limitations

The main strengths of our study encompass its real-world, population-based, case–control design, using a comprehensive nationwide database, with the application of a propensity scoring for matching the case and control groups, which enabled the identification of multiple clinical variables associated with complex patients. Findings remained consistent across sensitivity analyses, indicating that the observed associations were not driven by very old data, extreme utilization outliers, or age distribution, thereby supporting the robustness of our conclusions. The main limitation of the present study is its basis on a large nationwide HMO in a single country. As healthcare organizations vary considerably among countries, some of the variables identified might not be completely applicable to other locations or organizations. In addition, enrollment into the special program was subjective with a lack of complexity measurements and potential confounding. We indeed concentrated on clinical data, because the psychological data were limited in our database. Because we have extracted multiple variables retrospectively over a 10-year period for thousands of patients, we did not study clustering, disease severity, temporal alignment, and dose, duration and compliance of medication use. Selection bias due to clinician-driven enrollment may influence observed associations, and ORs should be interpreted as descriptive rather than causal. Although the TMCP program was implemented in all primary care outpatient clinics nationwide under standardized Ministry of Health guidelines, and cases were matched to controls by geographic region and socioeconomic status, residual facility-level variation cannot be completely excluded and is acknowledged as a limitation. Further research is suggested to confirm our findings with adaptation to other locations and organizations.

## 5. Conclusions and Future Aspects

The present comparative study, based on nationwide data, identified a series of clinical variables that were associated with real-life identification of the complex patients who were prioritized for inclusion in a special managed intervention program. The defined variables have potential uses for future efforts to validate selection criteria for managed programs. This might aid primary physicians in the management of complex patients and also assist in quality improvement efforts and prioritizing the utilization of healthcare resources for the complex patients who need them most. The importance of the findings is augmented by the increasing rates and complexity of patients in modern medicine. We currently follow meticulously the outcomes of the patients included in the special managed program and will report the findings in due time.

## Figures and Tables

**Table 1:** Matched demographics, physical measurements, and laboratory results of the case and control groups before enrollment.

		Cases (N = 2032)	Controls (N = 2032)	OR [95% CI]	*p* Value
Sex	Female	899 (44.2%)	899 (44.2%)	1.00 [0.88 to 1.13]	1.000
	Male	1133 (55.8%)	1133 (55.8%)	1.00 [0.88 to 1.13]	1.000
Age (mean ± SD, years)		64.8 ± 14.2	65.6 ± 13.8		0.078
Smoking status	Non-smokers	1504 (74.3%)	1497 (74.2%)	1.00 [0.87 to 1.16]	0.971
	Past smokers	81 (4.0%)	78 (3.9%)	1.04 [0.74 to 1.44]	0.872
	Smokers	440 (21.7%)	443 (21.9%)	0.99 [0.85 to 1.15]	0.879
Ethnic group	General	1218 (60.0%)	1266 (62.3%)	0.91 [0.80 to 1.03]	0.130
	Arab	517 (25.4%)	476 (23.4%)	1.12 [0.96 to 1.29]	0.144
	Jewish Ultra-orthodox	297 (14.6%)	290 (14.3%)	1.03 [0.86 to 1.23]	0.789
Region	Center	560 (27.6%)	538 (26.5%)	1.06 [0.92 to 1.22]	0.458
	Jerusalem	251 (12.4%)	249 (12.3%)	1.01 [0.83 to 1.22]	0.962
	North	781 (38.4%)	744 (36.6%)	1.08 [0.95 to 1.23]	0.243
	South	440 (21.7%)	501 (24.7%)	0.84 [0.73 to 0.98]	0.026
Socioeconomic status (1–20 scale)		8.27 ± 3.39	8.39 ± 3.36		0.274
Socioeconomic category	Low (0–6)	686 (36.4%)	649 (34.5%)	1.09 [0.95, 1.24]	0.146
	Middle (7–14)	1131 (59.9%)	1172 (62.3%)	0.91 [0.80, 1.04]	0.232
	High (≥15)	70 (3.7%)	65 (3.5%)	1.08 [0.77, 1.52]	0.538
Physical activity	>3 h weekly	76 (3.8%)	97 (4.9%)	0.77 [0.56 to 1.06]	0.103
	1–3 h weekly	291 (14.7%)	314 (15.9%)	0.91 [0.76 to 1.09]	0.310
	Occasionally	569 (28.7%)	612 (30.9%)	0.90 [0.78 to 1.03]	0.135
	None	1044 (52.7%)	954 (48.3%)	1.20 [1.05 to 1.36]	0.005
Weight (kg)		80.9 ± 19.5	81.2 ± 19.2		0.707
Height (cm)		165 ± 11	166 ± 11		0.435
BMI * (kg/m^2^)		29.4 ± 6.2	29.4 ± 6.0		0.948
BMI * category	Underweight (<18.5)	34 (1.7%)	31 (1.5%)	1.09 [0.65 to 1.84]	0.803
	Normal (18.5–24.9)	457 (22.6%)	452 (22.5%)	1.01 [0.86 to 1.17]	0.970
	Overweight (25–29.9)	695 (34.4%)	693 (34.5%)	0.99 [0.87 to 1.13]	0.947
	Obese (≥30)	836 (41.4%)	832 (41.4%)	1.00 [0.88 to 1.13]	0.974
Systolic blood pressure (mmHg)		132 ± 20	133 ± 19		0.361
Diastolic blood pressure (mmHg)		75.2 ± 10.8	75.5 ± 10.3		0.390
Hemoglobin (g/dL)		13.3 ± 2.0	13.6 ± 1.9		<0.001
Creatinine (mg/dL)		1.07 ± 1.09	1.02 ± 0.88		0.098
eGFR MDRD * (mL/min/1.73 m^2^, median [IQR])		54.0 (41.0–60.0)	52.0 (40.0–60.0)		0.132
Glucose (mg/dL)		147 ± 69	149 ± 68		0.290
Hemoglobin A1C (%)		7.50 ± 2.08	7.51 ± 2.03		0.846
Triglycerides (mg/dL)		165 ± 151	166 ± 169		0.838
HDL * Cholesterol (mg/dL)		45.6 ± 13.4	46.2 ± 12.8		0.145
LDL * Cholesterol (mg/dL)		98.8 ± 39.5	100.5 ± 39.5		0.159
Non-HDL * Cholesterol (mg/dL)		131 ± 47	133 ± 48		0.241
Albumin/Creatinine Ratio, median (IQR)		21.3 (8.3–95.5)	22.8 (7.4–81.3)		0.298
Macroalbuminuria		253 (12.5%)	219 (10.8%)	1.18 [0.97 to 1.43]	0.106
Microalbuminuria		834 (41.0%)	834 (41.0%)	1.00 [0.88 to 1.14]	1.000

* Abbreviations: BMI, body mass index; eGFR MDRD, estimated glomerular filtration rate modification of diet in renal diseases; HDL, high-density lipoprotein; LDL, low-density lipoprotein.

**Table 2:** Diagnoses in the case and control groups before enrollment.

Diagnosis	Cases (N = 2032)	Controls (N = 2032)	OR [95% CI]	*p* Value
**Respiratory Disorders**				
Bronchial asthma	237 (11.7%)	136 (6.7%)	1.84 [1.47 to 2.31]	<0.001
Chronic obstructive pulmonary disease	375 (18.5%)	314 (15.5%)	1.24 [1.05 to 1.46]	0.012
Obstructive sleep apnea	210 (10.3%)	158 (7.8%)	1.37 [1.10 to 1.71]	0.005
**Cardiovascular Disorders**				
Cardiomyopathy	79 (3.9%)	45 (2.2%)	1.79 [1.22 to 2.65]	0.002
Chronic heart failure	336 (16.5%)	278 (13.7%)	1.25 [1.05 to 1.49]	0.012
Myocardial infarction	265 (13.0%)	222 (10.9%)	1.22 [1.01 to 1.49]	0.042
Pulmonary embolism	50 (2.5%)	26 (1.3%)	1.95 [1.18 to 3.27]	0.007
Chronic atrial fibrillation	302 (14.9%)	204 (10.0%)	1.56 [1.29 to 1.90]	<0.001
**Rheumatic Disorders**				
Connective tissue disease	122 (6.0%)	74 (3.6%)	1.69 [1.25 to 2.30]	<0.001
Fibromyalgia	115 (5.7%)	62 (3.1%)	1.91 [1.38 to 2.66]	<0.001
Osteoporosis	339 (16.7%)	227 (11.2%)	1.59 [1.32 to 1.92]	<0.001
Osteoarthritis	741 (36.5%)	677 (33.3%)	1.15 [1.01 to 1.31]	0.038
**Allergic and Autoimmune Disorders**				
Hashimoto thyroiditis	218 (10.7%)	162 (7.9%)	1.39 [1.11 to 1.73]	0.003
Atopic dermatitis	201 (9.9%)	145 (7.1%)	1.43 [1.14 to 1.80]	0.001
Food allergy	385 (18.9%)	309 (15.2%)	1.30 [1.10 to 1.54]	0.001
**Gastrointestinal Disorders**				
Liver cirrhosis	33 (1.6%)	13 (0.6%)	2.56 [1.31 to 5.32]	0.004
Inflammatory bowel disease	43 (2.1%)	25 (1.2%)	1.74 [1.03 to 2.98]	0.037
**Neurologic Disorders**				
Cerebrovascular accident	233 (11.5%)	203 (10.0%)	1.17 [0.95 to 1.43]	0.14
Epilepsy	50 (2.5%)	24 (1.2%)	2.11 [1.27 to 3.61]	0.003
**Psychiatric Disorders**				
Attention-deficit hyperactivity disorder (ADHD)	43 (2.1%)	30 (1.5%)	1.44 [0.88 to 2.39]	0.155
Depression	135 (6.6%)	118 (5.8%)	1.15 [0.89 to 1.50]	0.298
Cognitive disorder	107 (5.3%)	68 (3.4%)	1.61 [1.17 to 2.22]	0.003
Deafness	507 (25.0%)	399 (19.6%)	1.36 [1.17 to 1.58]	<0.001
**Functional Impairment**				
Urine incontinence	496 (24.4%)	369 (18.2%)	1.46 [1.25 to 1.70]	<0.001
Previous fall	333 (16.4%)	222 (10.9%)	1.60 [1.33 to 1.93]	<0.001

**Table 3:** Chronic medications consumption by the case and control groups during the 10 years before the enrollment date. * Data are presented as numbers (%).

Variables *	Cases	Controls	OR [95% CI]	*p* Value
Long-acting insulins	825 (40.6%)	610 (30.0%)	1.59 [1.40 to 1.82]	<0.001
Short-acting insulins	485 (23.9%)	318 (15.7%)	1.69 [1.44 to 1.98]	<0.001
Sulfonylureas	484(23.8%)	423(20.8%)	1.19 [1.02 to 1.38]	<0.001
Sodium-glucose co-transporter 2 (Sglt2) inhibitors	489 (24.1%)	422 (20.8%)	1.19 [1.04 to 1.41]	<0.001
Glucagon-like peptide-1 (GLP-1) receptor analogs	615 (30.3%)	504 (24.8%)	1.32 [1.14 to 1.51]	<0.001
Selective beta-2 adrenoreceptor agonists, inhalation	643 (31.6%)	404 (19.9%)	1.87 [1.61 to 2.16]	<0.001
Anticholinergics	670 (33.0%)	446 (22.0%)	1.75 [1.52 to 2.02]	<0.001
Diuretics	935 (46.0%)	695 (34.2%)	1.64 [1.44 to 1.86]	<0.001
Glucocorticoids, inhalation	485 (23.9%)	302 (14.9%)	1.80 [1.53 to 2.11]	<0.001
Corticosteroids, systemic	1207 (59.4%)	1009 (49.7%)	1.48 [1.31 to 1.68]	<0.001
Short-acting opioids with or without non-opioid analgesics	1154 (56.8%)	956 (47.0%)	1.48 [1.30 to 1.68]	<0.001
Long-acting opioids (Buprenorphine)	140 (6.9%)	82 (4.0%)	1.76 [1.32 to 2.36]	<0.001
Long-acting opioids (Fentanyl)	107 (5.3%)	57 (2.8%)	1.93 [1.37 to 2.72]	<0.001
Benzodiazepines	1020 (50.2%)	778 (38.3%)	1.62 [1.43 to 1.84]	<0.001

**Table 4:** Emergency department (ED) visits and hospitalizations in the case and control groups during the 10-year period before the index date.

Variables	Cases, N (%)	Controls, N (%)	OR [95% CI]	*p* Value
Outpatient urgent care center visits	430 (21.2%)	230 (11.3%)	2.10 [1.76 to 2.51]	<0.001
Hospital emergency department visits	1733 (85.3%)	1544 (76.0%)	1.83 [1.56 to 2.16]	<0.001
Hospitalizations in internal medicine	1225 (60.3%)	851 (41.9%)	2.11 [1.86 to 2.39]	<0.001
Hospitalization for ≥4 days	679 (33.4%)	407 (20.0%)	2.00 [1.73 to 2.32]	<0.001
Inpatient evaluation/treatment in internal medicine	210 (10.3%)	122 (6.0%)	1.80 [1.42 to 2.30]	<0.001
Hospitalization in a geriatric-internal medicine department	61 (3.0%)	26 (1.3%)	2.39 [1.48 to 3.95]	<0.001
Inpatient rehabilitation in a general hospital	131 (6.4%)	88 (4.3%)	1.52 [1.14 to 2.03]	=0.003
Hospitalization in an intensive care unit	127 (6.3%)	85 (4.2%)	1.53 [1.14 to 2.05]	=0.004
Hospitalization in the geriatric rehabilitation center	42 (2.1%)	22 (1.1%)	1.93 [1.12 to 3.40]	=0.016

## Data Availability

All statistical analyses are available upon reasonable request. Because of ethical and privacy issues, patients’ data cannot be shared.

## Supplementary Materials

The following supporting information can be downloaded at: https://www.mdpi.com/article/10.3390/jcm15010202/s1. Table S1. Covariate Balance Before and After Propensity Score Matching. Table S2. Parsimonious Multivariable Conditional Logistic Regression. Table S3. Missing Data Sensitivity Analysis.
