# Supplementary material for: Clinical Variables Associated with Physician-Driven Inclusion in a Special Management Program for Complex Patients
Source: J Clin Med. 2025 Dec 26;15(1):202. doi: 10.3390/jcm15010202 (PMC12787282; doi:10.3390/jcm15010202)
Supplement: Supplementary file 1 [file jcm-15-00202-s001.zip › jcm-4027346-supplementary.pdf]

Supplementary Material – Clinical Variables Associated with Physician-Driven Inclusion in a Special Management Program for Complex Patients

Supplementary Table S1. Covariate Balance Before and After Propensity Score Matching

Covariate	SMD Before Matching	SMD After Matching
Age (years)	0.32	0.02
Sex (male %)	0.15	0.01
Socioeconomic status (score)	0.28	0.03
Geographic region	0.22	0.04
Smoking status	0.18	0.02
Physical activity	0.25	0.05
BMI (kg/m^2)	0.30	0.03
HbA1c (%)	0.27	0.02
Serum glucose (mg/dL)	0.26	0.03
LDL cholesterol (mg/dL)	0.21	0.04
eGFR ($mL/min/1.73\ m^2$)	0.29	0.03
Albumin/creatinine ratio	0.24	0.05

Supplementary Table S2. Parsimonious Multivariable Conditional Logistic Regression

Variable	OR (95% CI)	FDR-adjusted p-value
COPD	1.22 (1.05–1.42)	0.012
Fibromyalgia	1.65 (1.20–2.28)	<0.001
Osteoporosis	1.40 (1.18–1.66)	<0.001
**Polypharmacy (≥5 drug classes)**	1.78 (1.50–2.10)	<0.001
≥1 ED visit (10-year lookback)	1.90 (1.60–2.25)	<0.001
≥1 hospitalization (10-year lookback)	1.72 (1.48–2.00)	<0.001

Model: Conditional logistic regression within matched pairs; covariates selected based on clinical relevance and univariable significance.

Supplementary Table S3. Missing Data Sensitivity Analysis

Approach	OR for Polypharmacy	OR for ED Visit	OR for Hospitalization
Complete-case analysis	1.80 (1.52–2.12)	1.88 (1.58–2.23)	1.70 (1.46–1.98)
Missing-indicator	1.78 (1.50–2.10)	1.90 (1.60–2.25)	1.72 (1.48–2.00)
